# Microencapsulation of Mezcal Vinasse: Reduction of Undesirable Compounds Derived from the Production Process and Morphological Characterisation

**DOI:** 10.3390/foods15203633

**Published:** 2026-10-13

**Authors:** Alejandro Castrejon, Elisa Dufoo-Hurtado, Yuritzi Barbosa, Marcela Gaytán-Martínez, Aurea K. Ramírez-Jiménez

**Affiliations:** 1 School of Engineering and Sciences, Tecnologico de Monterrey, Avenida Eugenio Garza Sada 2501 Sur, Monterrey 64849, NL, Mexico; a00835414@tec.mx (A.C.); elisa.dufoo@tec.mx (E.D.-H.); 2 División de Ingenierías, Universidad Anáhuac Querétaro, Cto. Universidades I, Fracción 2, Zibatá, Querétaro 76246, QRO, Mexico; yuritzi.barbosaa@anahuac.mx; 3 Posgrado en Ciencia y Tecnología de los Alimentos, Research and Graduate Studies in Food Science, School of Chemistry, Universidad Autónoma de Querétaro, Centro Universitario, Santiago de Querétaro Qro 76010, CP, Mexico; marcelagaytanm@yahoo.com.mx

**Keywords:** mezcal vinasse, 5-(hydroxymethyl)furfural reduction, spray drying, corn starch, particle morphology

## Abstract

Mezcal vinasse contains potentially valuable compounds but also 5-(hydroxymethyl)furfural (HMF), which limits its utilization as a food ingredient. This study evaluated a sequential process comprising HMF mitigation with activated carbon and laccase under a central composite design, followed by corn starch microencapsulation through spray drying using a 15-run Box–Behnken design. The best observed HMF-treatment condition, consisting of 0.01 g activated carbon/100 mL, 873 µL laccase/100 mL, and pH 4.5, achieved a 76.69% HMF reduction while retaining 72.30% of the phenolic compounds. The quadratic HMF model showed R^2^ = 0.921 and adjusted R^2^ = 0.850. For microencapsulation, the best condition (140 °C inlet temperature, 50 V airflow, and 10 mL/min solution flow) produced the lowest water activity (0.1306), showed no resolvable low-temperature differential scanning calorimetry (DSC) endotherm, and exhibited comparatively smooth, low-defect morphology. Across the formulations, FTIR fingerprint-region correlations ranged from 0.9612 to 0.9996, and the first two principal components explained 91.1% of spectral variability. Median particle-domain diameter ranged from 4.331 to 5.808 µm, circularity from 0.556 to 0.599, and solidity from 0.837 to 0.864. Overall, the process reduced HMF while preserving phenolic compounds and produced a physically stabilized vinasse powder, allowing for a first step into the revalorization of mezcal vinasse.

## 1. Introduction

Mezcal is a traditional beverage of Mexico, deeply rooted in the country’s cultural heritage and linked to the history of agave utilization since pre-Hispanic times. Beyond its role as an alcoholic beverage, mezcal represents a biocultural product shaped by regional traditions, local biodiversity, and artisanal knowledge [1]. The term mezcal derives from the Nahuatl word mexcalli, meaning “cooked agave”, reflecting the historical importance of agave processing in Mesoamerican societies [1]. Today, mezcal is produced from numerous agave species across the protected Denomination of Origin region, where variations in raw materials, environmental conditions, and production techniques contribute to its diversity and sensory characteristics [1]. Consequently, mezcal has evolved from a regional traditional spirit into a globally recognized beverage while maintaining a strong connection to Mexican identity and cultural traditions. This growing international recognition has also stimulated scientific interest in understanding both the beverage itself and its production chain and associated by-products [2,3,4].

The growing production of mezcal has been accompanied by an increase in the generation of agro-industrial by-products, among which vinasse represents one of the most abundant and environmentally challenging residues [3]. Vinasse is the liquid effluent generated during the distillation process and is characterized by a low pH, high organic load, and considerable concentrations of dissolved organic matter, which makes disposal and treatment difficult [5]. Previous studies have demonstrated that mezcal vinasse contains residual carbohydrates, dietary fiber, phenolic compounds, antioxidants, and other fermentation- and processing-derived metabolites with potential industrial applications [6].

Among the compounds identified in agave vinasses, 5-(hydroxymethyl)furfural (HMF) and furfural have attracted particular attention due to their implications for food and ingredient applications. Both compounds are generated through the thermal degradation of carbohydrates under acidic conditions and are commonly formed during the cooking and distillation stages of mezcal production [7]. Although HMF and furfural naturally occur in a wide variety of thermally processed foods and beverages, elevated concentrations have raised concerns because of their potential biological effects. Previous studies have reported that HMF may undergo metabolic activation into reactive intermediates capable of inducing cytotoxic, genotoxic, and mutagenic responses under experimental conditions, while furfural has also been associated with adverse cellular effects at high exposure levels [7,8,9]. Consequently, their presence represents an important obstacle for the direct utilization of mezcal vinasse as a functional food ingredient, highlighting the need for strategies capable of reducing their concentration while preserving the beneficial compounds naturally present in the matrix.

While the reduction of HMF and furfural is an important step toward the safe utilization of mezcal vinasse, preserving its beneficial constituents is equally important. A recent characterization of mezcal vinasse demonstrated that this by-product contains considerable amounts of dietary fiber, phenolic compounds, flavonoids, antioxidant molecules, and other bioactive constituents with promising functional potential [6]. However, many of these compounds are susceptible to degradation during processing and storage because of oxidation, moisture uptake, and thermal exposure, which may reduce their biological activity and technological value [10,11]. Therefore, successful valorization strategies should simultaneously decrease the concentration of undesirable thermal degradation products while protecting and stabilizing the remaining bioactive compounds, maximizing their potential for subsequent food applications.

Among the technologies available for the stabilization of bioactive compounds, microencapsulation has emerged as an effective approach for protecting sensitive molecules against environmental degradation while improving storage stability, handling characteristics, and facilitating their incorporation into food formulations [12]. Spray drying is particularly attractive because of its scalability, relatively low operating cost, and broad industrial applicability for converting liquid matrices into stable powdered ingredients [12,13,14]. The selection of the encapsulating material is fundamental to achieving high encapsulation efficiency and maintaining the stability of the encapsulated compounds. In this regard, corn starch represents a promising wall material due to its favorable film-forming capacity, thermal stability, and ability to effectively protect bioactive compounds during processing and storage [15].

The development of this sequential process requires linking chemical treatment performance with the physicochemical characteristics of the final powder. In particular, reducing HMF while retaining phenolic compounds addresses the composition of the liquid feed, whereas water activity, thermal behavior, and particle morphology provide complementary information about the powder obtained after drying. This study addresses the integration of these two stages in mezcal vinasse valorization, focusing on the balance between HMF mitigation and phenolic preservation and on the influence of spray-drying conditions on powder characteristics.

Accordingly, the objective of this study was to develop and evaluate a sequential treatment and microencapsulation strategy for mezcal vinasse. First, a central composite design was used to assess the effects of activated carbon addition, laccase addition, and pH on HMF reduction and total phenolic retention, with HMF quantified by high-performance liquid chromatography coupled to diode-array detection (HPLC-DAD). Subsequently, a Box–Behnken design was used to evaluate the effects of inlet temperature, airflow setting, and feed rate on spray-drying microencapsulation with corn starch. The resulting powders were characterized by water-activity measurements, Fourier-transform infrared spectroscopy (FTIR), differential scanning calorimetry (DSC), and scanning electron microscopy (SEM), complemented by image analysis to assess particle size and morphology. This approach provided an experimental basis for evaluating HMF mitigation, phenolic preservation, and the physicochemical characteristics of vinasse-derived powders.

## 2. Materials and Methods

### 2.1. Materials

Corn starch (Corrupac, Adisa, Mexico State, Mexico), activated carbon (Hycel, Guadalajara, Mexico), laccase from *Aspergillus* sp. (Merck, Darmstadt, Germany), and all analytical-grade reagents were used throughout the study.

### 2.2. Mezcal Vinasse Collection and Sample Preparation

Mezcal vinasse was collected from the mezcal distillery “La Cascada,” in Malinalco, State of Mexico, at an approximate elevation of 1750 m above sea level. This vinasse originated from the production of mezcal using *Agave angustifolia*. Samples were collected directly after the distillation process and transported to the laboratory under refrigerated conditions. Upon arrival, the samples were stored at −80 °C until further use.

Before experimentation, vinasse samples were thawed at 4 °C under standard laboratory conditions and homogenized. When necessary, samples were filtered to remove suspended solids. Aliquots were prepared according to each experimental treatment and analyzed immediately.

### 2.3. Experimental Strategy

The study was carried out in two sequential optimization stages. In the first stage, mezcal vinasse was subjected to different treatment conditions in which the pH was fixed, activated carbon was added, and specific volumes of lacasse were added to the vinasse. The specific values for each variable added can be seen in Table 1; these treatments were aimed at reducing HMF and furfural content while preserving phenolic compounds.

The laccase stock solution exhibited a volumetric activity of 28.65 U mL^−1^ under the assay conditions.

In the second stage, the optimized treated vinasse was used as feed material for spray-drying microencapsulation with corn starch as wall material. Spray-drying conditions were optimized to obtain powders with suitable physicochemical properties, including low moisture content, low water activity, adequate thermal behavior, and desirable particle morphology.

### 2.4. Optimization of HMF Reduction

#### 2.4.1. Experimental Design

The reduction of HMF in mezcal vinasse was optimized using a Central Composite Design generated with Minitab Statistical Software (Minitab Version 21, LLC, 2021, State College, PA, USA). The independent variables evaluated were pH, lacasse concentration, and active carbon concentration. The levels for each factor are shown in Table 1. The response variables were HMF concentration (%) and total phenolic content (%) after treatment on a dry basis. The objective of the optimization was to minimize HMF concentrations while maintaining the highest possible retention of phenolic compounds. The experimental design included 20 runs, with 8 factorial points, 6 axial points, and 6 center points. All treatments were performed in triplicate, and the order of the runs was randomized to minimize systematic error.

#### 2.4.2. Vinasse Treatment Procedure

For each experimental run, 50 mL of mezcal vinasse was placed in a 50 mL Falcon tube. The pH was adjusted to the value established by the experimental design (Table 1) using HCl (0.1 M) or NaOH (0.1 M). Activated carbon and laccase were added at the concentrations described in Table 1 according to the corresponding treatment condition [6].

Samples were incubated at 37 °C for 30 min under agitation conditions using a water bath (SWB 15S Water Bath, Thermo Fisher Scientific, Winchester, VA, USA). After treatment, samples were centrifuged at 1000× *g* for 3 min at 25 °C and filtered to remove suspended material and activated carbon residues. The treated vinasse was then collected and stored at −20 °C until further analysis.

#### 2.4.3. HMF and Quantification by HPLC-DAD

HMF was quantified by high-performance liquid chromatography coupled to diode array detection (HPLC-DAD). Separation was achieved using a Zorbax Eclipse XDB-C18 column (250 mm × 4.6 mm, 5 µm; Agilent Technologies, Santa Clara, CA, USA), which was maintained at a temperature of 30 °C. The mobile phase consisted of acetonitrile and water in an 80:20 (*v*/*v*) ratio, delivered at a 1.0 mL/min flow rate. Each sample had an injection volume of 20 µL. Detection was carried out with the DAD at 285 nm for HMF. The total run time for each analysis was 15 min.

### 2.5. Spray-Drying Optimization

#### 2.5.1. Experimental Design

Spray-drying conditions were optimized using a Box–Behnken design generated with MiniTab Statistical Software. The independent variables were inlet temperature, flow rate, and air volume. The levels evaluated for each factor are shown in Table 2.

The response variables were water activity, thermal properties, and morphological characteristics of the resulting powders. The design consisted of 15 experimental runs. All experiments were performed in duplicate, and the order of the runs was randomized.

#### 2.5.2. Feed Preparation

The optimized treated vinasse obtained from Section 2.4 was used as the core material for microencapsulation. Corn starch was added as wall material at 20% (*m*/*v*) and dispersed in the vinasse under constant agitation. The feed suspension was maintained under agitation until spray drying to prevent sedimentation and ensure homogeneity. Corn starch was selected as a rich carbohydrate carrier to evaluate the feasibility of producing starch/vinasse powders. Its concentration and its use as the sole carrier were maintained throughout the experimental design, allowing the influence of spray-drying conditions to be evaluated without introducing differences in carrier composition. Comparison of alternative carriers or carrier blends was outside the scope of this study.

#### 2.5.3. Spray-Drying Procedure

Spray drying was performed using a laboratory-scale spray dryer (Mini Spray Dryer B-290, BÜCHI, Flawil, Switzerland). The feed suspension was dried under the conditions established by the experimental design. The resulting powders were collected from the cyclone separator, weighed, and stored in airtight containers protected from light until characterization. Water activity was measured using a water activity meter (Aqua LAB, Decagon Devices, Pullman, Washington, DC, USA) at 25 °C.

### 2.6. Physicochemical Characterization of Microencapsulated Powders

#### 2.6.1. Fourier-Transform Infrared Spectroscopy (FTIR)

Fourier-transform infrared spectroscopy (FTIR) was used to identify functional groups and possible interactions between vinasse components and corn starch. Spectra were obtained using a Spectrum Two FTIR spectrometer (PerkinElmer Inc., Waltham, MA, USA) equipped with an attenuated total reflectance accessory.

#### 2.6.2. Differential Scanning Calorimetry

Thermal properties of the powders were evaluated by DSC (Q2000: TA INSTRUMENTS). Approximately 3 mg of sample was placed in aluminum pans and heated from 30 to 160 °C under a nitrogen atmosphere.

Thermograms were analyzed to identify endothermic or exothermic transitions, including water loss, starch-related thermal events, and possible matrix interactions.

#### 2.6.3. Scanning Electron Microscopy and Particle Size Analysis

The morphology of the spray-dried powders was examined by scanning electron microscopy using a high-vacuum SEM (JSM-6060LV, JEOL Ltd., Tokyo, Japan). Powder samples were mounted on bronze specimen holders using double-sided carbon tape and coated with a thin layer of gold prior to observation. Micrographs were acquired at an accelerating voltage of 12 kV and at magnifications ranging from 50× to 2500×. Images obtained at 1000× were used for particle-size and shape analysis, whereas micrographs acquired at 2500× were used to evaluate surface morphology.

Particle dimensions were estimated from scale-calibrated SEM images using ImageJ (Version 1.54t) software. The scale bars included in the micrographs were used for spatial calibration, and at least 50 clearly delineated particle domains were measured per experimental condition. Because the particle-size distributions were asymmetric and several particles exhibited partial fusion or agglomeration, particle size was expressed as the median equivalent circular diameter (D_50_), together with D_10_ and D_90_. Particle-size distribution width was described using the span, which reflects the relative breadth of the particle-size distribution based on the D_10_, D_50_, and D_90_ values. Particle shape compactness was assessed using solidity, calculated from ImageJ measurements by comparing the projected particle area with the area of its convex hull; values closer to 1 indicate more compact particles with fewer surface indentations or irregularities.

Morphology was qualitatively evaluated according to surface smoothness, wrinkling, shrinkage or collapse, agglomeration, fissures, and overall microcapsule integrity.

### 2.7. Statistical Analysis

All experiments were performed in triplicate, and results were expressed as mean ± standard deviation. Response surface methodology was used to evaluate the effect of the independent variables on the selected response variables. Regression models were fitted using MiniTab Statistical Software, and model significance was assessed by analysis of variance (ANOVA).

Linear, quadratic, and interaction effects were considered significant at *p* < 0.05. When appropriate, differences between treatments were analyzed using Tukey’s test at a significance level of *p* < 0.05.

Principal component analysis (PCA) was performed to visualize multivariate patterns among the FTIR spectral variables and to identify the main sources of variability among the spray-dried formulations. The analysis was conducted using Minitab Statistical Software, and the first principal components were retained based on the proportion of total variance explained.

Optimization was performed using a desirability function to identify the conditions that minimized HMF content while maximizing phenolic retention and improving powder stability. Graphical response surface plots and contour plots were generated to visualize the effect of the experimental factors on the response variables.

## 3. Results

### 3.1. HMF Reduction

The central composite design shown in Table 1 was applied to evaluate the combined effect of activated carbon, laccase, and pH on HMF mitigation and phenolic compound preservation in mezcal vinasse. The treatments resulted in an HMF reduction range from 74.8% to 93.9%, showing that the selected processing variables were suitable to modulate the concentration of HMF. Phenolic retention also varied considerably among treatments, from 2.9% to 72.3%.

The quadratic model fitted for HMF reduction showed adequate predictive performance, with an R^2^ of 0.921 and an adjusted R^2^ of 0.850.

According to the PCA, laccase concentration was the principal component influencing HMF reduction (*p* < 0.05). However, its effect was negative, indicating that increasing laccase concentration did not favor HMF removal under the evaluated conditions. This trend is illustrated in Figure 1, where higher predicted HMF reductions occur at lower laccase levels. The contour plots describe how predicted HMF reduction varies with the displayed factors while the remaining factor is held constant. Although activated carbon and pH were not the principal components influencing HMF reduction, their contribution to the response surface suggests that HMF reduction was modulated by the combined physicochemical conditions of the treatment.

The contour plots indicated that HMF reduction was favored under conditions of low laccase concentration and acidic pH, with activated carbon contributing differently depending on the fixed level of the third factor (Figure 1).

The response surface plots for phenolic retention showed that higher laccase levels tended to favor phenolic preservation, whereas lower laccase levels were associated with stronger HMF reduction (Figure 2).

Figure 2 illustrates the predicted phenolic response to paired changes in activated carbon addition, laccase addition, and pH, with the third factor held constant in each plot. The curved surfaces indicate that the predicted response varies across the experimental domain.

Under this constrained optimization approach, the most suitable predicted condition was approximately 0.0124 g of activated carbon, 746.4 µL of laccase, and pH 2.88. This condition predicted an 80.04% reduction in HMF while retaining approximately 60.11% of the phenolic compounds.

Among the experimental treatments, Run 9 represented the best observed compromise, with a 76.69% reduction in HMF and 72.30% phenolic retention. Thus, Run 9 can be considered the best experimental condition.

### 3.2. Spray-Drying Microencapsulation and Characterization

With these conditions taken into consideration, the vinasse used for spray drying was treated under the conditions corresponding to Run 9 (Table 1). For the microencapsulation, 15 runs (referred to as EXP) were conducted; the conditions are described in Table 2.

#### 3.2.1. Water Activity

Water activity varied among the experimental spray-drying conditions, ranging from 0.13 to 0.39. The lowest value was observed for EXP14, followed by EXP8 and EXP6, whereas EXP2 exhibited the highest water activity. The run-level mean was 0.26 ± 0.07, with a median of 0.28. All formulations presented water-activity values below 0.40, and 14 of the 15 experimental runs remained below 0.35.

Three formulations, EXP14, EXP8, and EXP6, exhibited water-activity values below 0.20, whereas EXP13 and EXP4 showed values between 0.20 and 0.25. In contrast, EXP2 was clearly differentiated from the remaining formulations, with an aw value of 0.39.

The broad range of water-activity values indicates that the experimental spray-drying conditions affected the availability and binding state of residual water within the starch/vinasse mix.

#### 3.2.2. FTIR Analysis

The FTIR spectra of the 15 spray-dried formulations exhibited a common absorption pattern, indicating that the powders retained a broadly similar chemical composition. Figure 3 presents selected formulations illustrating contrasting structural and hydration-related characteristics within the common FTIR absorption pattern. EXP9 exhibited the highest apparent short-range order index, whereas EXP15 and EXP10 showed the highest and lowest hydration-sensitive index values, respectively. EXP14 was included to relate these spectral features to the formulation combining the lowest water activity with comparatively smooth particle surfaces. The spectra were characterized by a broad O–H stretching band between 3280 and 3305 cm^−1^, C–H stretching at 2928–2937 cm^−1^, a weak carbonyl-associated shoulder at approximately 1709–1723 cm^−1^, a reproducible band at 1587–1590 cm^−1^, and marked carbohydrate-associated absorptions within the 1200–900 cm^−1^ region. The principal carbohydrate maximum was located between 1012 and 1017 cm^−1^.

No formulation-specific strong absorption band was detected. Pairwise correlations calculated over the normalized fingerprint region ranged from 0.9612 to 0.9996, with a mean of 0.9909, confirming the high overall similarity of the formulations. PCA nevertheless differentiated the powders according to variations concentrated mainly between approximately 990 and 1077 cm^−1^.

The apparent short-range order index R_1047/1022_ varied from 0.682 to 0.805. The highest value was observed for EXP9, followed by EXP7, EXP12, and EXP13, whereas EXP15 and EXP2 presented the lowest values. The hydration-sensitive R_995/1022_ index varied more markedly, from 0.640 in EXP10 to 0.959 in EXP15. The contrasting behavior of these ratios demonstrated that the processing conditions produced distinct combinations of local carbohydrate organization and hydration-related structure.

The O–H stretching maximum also varied among formulations. EXP1, EXP2, EXP3, EXP5, EXP10, and EXP15 displayed lower-frequency maxima, approximately 3280–3284 cm^−1^, whereas EXP6, EXP8, EXP11, EXP13, and EXP14 exhibited values near 3300–3305 cm^−1^. These differences indicate variation in the hydrogen-bonding environment of the powders.

#### 3.2.3. Differential Scanning Calorimetry (DSC)

DSC revealed marked differences in the thermal behavior of the spray-dried powders. Most formulations displayed a broad endothermic transition between approximately 75 and 112 °C. A resolvable event was identified in 11 of the 15 experimental runs, with peak temperatures ranging from 92.3 to 104.6 °C and a descriptive mean of 98.3 ± 3.5 °C. The apparent enthalpy associated with this transition varied from 0.91 to 11.49 J g^−1^, indicating substantial differences in the amount of energy involved in water release or matrix reorganization among the experimental conditions.

Figure 4 compares the starch control with selected powders exhibiting contrasting thermal responses. The starch control provides a reference for the high-temperature endothermic event near 157.5 °C, whereas EXP1 illustrates a pronounced low-temperature transition and has the highest apparent enthalpy among the formulations. EXP14 represents the absence of a resolvable low-temperature endotherm, consistent with its comparatively low water activity. EXP15 provides a change near the upper limit of the temperature scan. Together, these profiles illustrate the range of thermal behavior observed in the starch–vinasse powders.

EXP1 and EXP10 presented the highest apparent enthalpy values, 11.49 and 9.52 J g^−1^, respectively, whereas EXP4 and EXP13 showed weak but distinguishable events with values below 1.1 J g^−1^. No resolvable low-temperature endothermic transition was detected in EXP6, EXP8, or EXP14.

The starch control showed a markedly different thermal profile. It did not display the broad low-temperature endotherm characteristic of most microencapsulated samples; instead, it exhibited a sharp high-temperature endothermic event with a minimum near 157.5 °C.

#### 3.2.4. Morphology and Particle Size by Scanning Electron Microscopy (SEM)

Scanning electron microscopy revealed a heterogeneous population of rounded, polyhedral, lobed, and irregular particle domains, frequently arranged in partially fused agglomerates. Quantitative analysis of 750 clearly delineated domains from the 1000× micrographs showed that the median apparent particle-domain diameter ranged from 4.331 to 5.808 µm, with a run-level mean of 4.890 ± 0.401 µm. EXP13 presented the largest median diameter, followed by EXP11, EXP8, EXP15, and EXP5, whereas EXP1 exhibited the smallest median value.

The width of the particle-domain distributions also varied among the experimental conditions. Span values ranged from 0.849 to 1.395. EXP10 showed the broadest distribution and the highest D_90_, consistent with the coexistence of small particles and large fused structures. In contrast, EXP12, EXP1, and EXP2 exhibited comparatively narrower distributions.

The mean circularity values ranged from 0.556 to 0.599, indicating that the particle domains differed from perfect spherical geometry. EXP14, EXP5, and EXP13 showed the highest circularity, whereas EXP8, EXP7, and EXP12 presented the most irregular projected shapes. Solidity varied from 0.837 to 0.864, indicating that most domains remained relatively compact despite the presence of folds and surface indentations.

At higher magnification, differences in the surface became evident. EXP5, EXP13, EXP14, and EXP15 displayed comparatively smooth surfaces and low frequencies of pronounced defects. Conversely, EXP4, EXP7, EXP8, EXP10, and EXP12 exhibited greater wrinkling, folding, fissuring, and localized crater-like structures; the results of these contrasting structures can be seen in Figure 5. These results indicate that the spray-drying conditions affected not only the apparent dimensions of the particles but also their degree of fusion, geometric regularity, and surface deformation.

## 4. Discussion

### 4.1. HMF Reduction and Preservation of Phenolic Compounds

HMF reduction ranged from 74.76 to 93.89%, whereas phenolic retention ranged from 2.92 to 72.30%. In particular, the treatment with the highest observed HMF reduction was Run 19, accompanied by very low phenolic retention. Run 9 produced a more balanced response, with 76.69% HMF reduction and 72.30% phenolic retention. The desirability-based predicted condition followed the same logic, with a lower predicted HMF reduction (80.04%) and a higher predicted phenolic retention (60.11%).

Activated carbon is effective for removing furanic inhibitors, but its adsorption is not selective for HMF. Mamaní Arminda et al. [16], working with a lignocellulosic hydrolysate, reported simultaneous removal of 64% HMF and 55% total phenolic compounds under optimized activated-carbon conditions. The study illustrates that adsorption can decrease an undesirable furan while also depleting compounds that are desirable for subsequent functional applications. The very low phenolic retention observed in some of the present treatments is therefore consistent with competitive adsorption of chemically diverse aromatic and polar compounds onto the carbon surface.

Laccase showed an apparently counterintuitive effect in the fitted HMF model: increasing laccase addition did not improve HMF reduction, and the highest predicted reductions occurred at lower laccase levels. This result does not imply that laccases are unable to transform HMF. Recent studies have demonstrated laccase-mediated HMF oxidation, but those systems were specifically configured for that reaction. Araya et al. [17] optimized the oxidation of HMF using Trametes versicolor laccase with TEMPO as a redox mediator, while Wei et al. [18] used a CotA laccase/TEMPO/catalase system in which oxygen availability was actively supported. In contrast, the present vinasse treatment used laccase directly in a chemically complex acidic matrix and no redox mediator. Therefore, the negative laccase coefficient in the fitted HMF reduction model should be interpreted within the experimental domain were there was not an added redox mediator. Within this realm, increasing laccase addition was associated with lower HMF reduction. This association does not establish the underlying mechanism or imply that laccase would behave similarly under other reaction conditions.

The broad range of phenolic retention requires consideration of both treatment-related differences and experimental variability. Across the design, changes in activated carbon addition, laccase addition, and pH may have influenced the balance between phenolic adsorption and transformation within the vinasse matrix. The simultaneous removal of HMF and phenolic compounds reported for activated-carbon treatment in another complex substrate supports the possibility of limited selectivity [16]. Total phenolic measurements do not distinguish among compound-specific adsorption, oxidation, and other transformations, so the mechanisms underlying the observed retention cannot be conclusively assigned.

The results also indicate that maximizing HMF removal and maximizing phenolic retention were competing objectives within the evaluated experimental domain. Accordingly, the treatment was selected as a compromise between these responses rather than as a condition that simultaneously maximized both. The present findings do not establish that an additional pretreatment is necessary to improve this balance. Further work could evaluate the sequence of carbon and enzyme addition or an additional separation step, but any benefit would need to be demonstrated through direct comparison with the current procedure.

From a process-development perspective, the quadratic HMF model (R^2^ = 0.921; adjusted R^2^ = 0.850) was sufficiently informative to identify a useful region within the experimental domain. Overall, this first stage demonstrates a general principle for vinasse valorization where detoxification must be evaluated together with retention of the chemical fraction that gives the by-product its intended functional value.

### 4.2. Water Activity of the Spray-Dried Microencapsulates

The spray-dried powders showed consistently low water activity, with values between 0.1306 and 0.3873. All 15 formulations were below 0.40, indicating that spray drying effectively reduced the availability of water in the vinasse–starch matrix. The differences among experiments are nevertheless relevant because they show that the final water state was sensitive to the drying conditions rather than being determined only by the presence of the starch carrier.

Comparable process effects have been reported for other spray-dried food systems. Tao et al. [19] observed a progressive decrease in water activity as inlet temperature increased during production of grapeseed-oil microcapsules, reaching an aw of 0.28 at 180 °C. Arepally and Goswami [20] likewise reported water-activity values of approximately 0.52 to 0.33 in spray-dried probiotic powders as inlet temperature and gum-Arabic concentration were varied. These studies support the general interpretation that drying intensity and formulation influence the amount and availability of residual water.

The starch carrier is also likely to contribute to the final water state. Deng et al. [21] compared five wall materials for spray-dried bitter-gourd powder and found that all wall materials reduced water activity relative to the nonencapsulated powder; starch was one of the effective carriers. This agrees with the present use of corn starch as a matrix capable of producing a low-aw powder. Consequently, the present water-activity results establish that the drying process produced powders with low water availability and identify EXP14 as particularly favorable for this response, while long-term storage experiments would be required to demonstrate the practical stability of the optimized powder.

### 4.3. FTIR Analysis

The FTIR spectra of the spray-dried formulations exhibited a common absorption profile dominated by the carbohydrate-rich matrix (Figure 3). The broad O–H stretching band at 3280–3305 cm^−1^, C–H stretching at 2928–2937 cm^−1^, and intense absorptions within 1200–900 cm^−1^ were retained across the experimental conditions. Together with the high fingerprint-region correlations (0.9612–0.9996), these observations indicate substantial similarity in the dominant functional group regions. Nevertheless, spectral similarity should not be interpreted as evidence of identical composition or complete chemical preservation, because overlapping absorptions and the contribution of the starch carrier may obscure changes in less abundant vinasse constituents.

The 1200–900 cm^−1^ region is sensitive to local molecular organization. Lu et al. [22] demonstrated that infrared band ratios in this region can track changes in short-range starch organization during retrogradation, while An et al. [23] combined infrared and Raman spectroscopy to show that changes in nearby carbohydrate bands reflect evolution of local ordered structures. PCA complemented these observations by summarizing spectral variation that was less apparent from the overall similarity of the spectra. The first two principal components explained 91.1% of the analyzed spectral variability, with differentiation concentrated mainly around 990–1077 cm^−1^. This pattern is consistent with differences in the relative contributions of carbohydrate-associated bands, but PCA alone does not establish the molecular mechanism responsible.

Wang et al. [24] showed that acetylation changes starch structure and that the response depends on the original crystalline pattern, demonstrating that chemical modification and molecular organization are interdependent. Consequently, the differences among EXP9, EXP7, EXP12, EXP13, and the lower-index formulations may reflect different degrees of local ordering, hydration, or packing generated during rapid dehydration.

The O-H maximum shifted over a range of approximately 25 cm^−1^ among formulations, from about 3280–3284 to 3300–3305 cm^−1^. These shifts may reflect changes in hydration and intermolecular association during drying, but they cannot uniquely identify starch–phenolic interactions or demonstrate the formation of new covalent bonds. Similarly, the weak shoulder at 1709–1723 cm^−1^ is compatible with carbonyl-containing constituents, although its presence alone does not establish their identity or concentration. Porras-Saavedra et al. [25], using starch-containing wall systems for spray-dried microcapsules, likewise observed preservation of characteristic starch FTIR bands in the resulting particles, supporting the use of FTIR primarily as evidence of matrix integrity and intermolecular environment rather than as proof of a new covalent structure.

Overall, the FTIR findings support broadly similar functional-group profiles accompanied by variations consistent with local carbohydrate organization and hydration. These observations complement the DSC and morphological results without independently demonstrating crystallinity, encapsulation efficiency, or chemical stability. This is a favorable outcome for process optimization: the drying conditions altered matrix organization without evidence of major formation or loss of dominant functional groups.

### 4.4. DSC Analysis

The broad endothermic transitions detected in most microencapsulated powders, centered at approximately 98 °C, are most consistently interpreted as events dominated by the desorption and evaporation of water retained within the carbohydrate matrix.

The large variation in apparent enthalpy among the experimental runs therefore suggests that the spray-drying conditions affected either the quantity of water retained by the powder, the strength of water–matrix interactions, the structural order of the wall material, or a combination of these phenomena. Higher ΔH values, such as those observed for EXP1 and EXP10, indicate that more energy was required to complete the endothermic process.

The dominant DSC feature of the microencapsulated powders was a broad endothermic transition between approximately 75 and 112 °C, with resolvable peak temperatures of 92.3–104.6 °C in 11 of the 15 formulations. The broad shape, its position near the boiling region of water, and the large variation in apparent enthalpy (0.91–11.49 J g^−1^) are most consistent with an event dominated by release of water associated with the carbohydrate-rich matrix, with possible overlap from matrix reorganization. Comparable thermal behavior has been described in other spray-dried food matrices. Barra et al. [26] reported broad endothermic events associated with dehydration in alginate/gum-Arabic microcapsules, whereas AlYammahi et al. [27] combined DSC with complementary thermal analyses to investigate the thermal behavior of camel-milk powders containing date extract. These studies provide context for interpreting the broad endotherm observed in the present starch–vinasse powders as potentially involving water release. However, overlapping changes in matrix organization may also contribute to the measured heat flow; therefore, this event cannot be assigned exclusively to water evaporation on the basis of DSC alone.

The internal agreement with the water-activity data strengthens this interpretation. EXP6, EXP8, and EXP14, which had the lowest water activities, showed no resolvable low-temperature endothermic event. Conversely, EXP1 and EXP10 had the largest apparent enthalpies, indicating that substantially more energy was involved in the transition. Because water activity reflects the thermodynamic availability of water rather than its total quantity, the two measurements are not interchangeable. Nevertheless, their qualitative agreement suggests that the drying conditions changed both the amount of residual water and/or the strength with which water was associated with the starch–vinasse matrix.

The starch control exhibited a different event, with a sharp endothermic minimum near 157.5 °C. Starch-based encapsulating systems can show higher-temperature endothermic transitions depending on chemical modification and matrix composition. García-Gurrola et al. [28], for example, reported endothermic transitions between approximately 142 and 174 °C for spray-dried microcapsules prepared with esterified sorghum starches, with the phosphorylated system reaching 173.89 °C. This makes a starch-associated transition plausible for the present control.

Overall, DSC provides a complementary view of the microencapsulation process. The low-temperature events primarily distinguish formulations according to water–matrix interactions, whereas the high-temperature signal of the starch control points to a separate starch-associated transition that was only partially captured by the selected scan range.

### 4.5. SEM Morphology and Apparent Particle Size

The SEM analysis showed that the spray-dried vinasse powders formed micrometric structures, but they were not uniformly spherical primary particles. Instead, the micrographs contained rounded, polyhedral, lobed, and irregular domains, often present in partially fused agglomerates. The median apparent particle-domain diameter varied only within a relatively narrow interval (4.331–5.808 µm), while the span, circularity, and solidity revealed clearer differences in particle architecture.

Irregularity, indentation, wrinkling, and partial collapse are common outcomes of rapid droplet drying. Both et al. [29] showed that the morphology of spray-dried maltodextrin/whey-protein particles depended strongly on both composition and drying conditions. In a starch-based system that is closer to the present work, García-Gurrola et al. [28] reported spray-dried esterified-starch microcapsules with irregular shapes, indentations, and concavities, with particle dimensions in the micrometer range.

Combining starch with an additional carrier represents a possible strategy for modifying particle morphology. Porras-Saavedra et al. [25] obtained spherical microcapsules using Sechium edule starch in combination with gum-Arabic and whey protein concentrate to encapsulate cinnamon oleoresin. Their findings support the evaluation of multicomponent wall materials, although differences in starch source, encapsulated material, and processing conditions prevent direct extrapolation to the present vinasse system. A co-carrier could potentially improve particle regularity, but greater morphological uniformity cannot be assumed without a direct comparison. The present study therefore establishes a single carrier baseline against which future starch-based blends could be evaluated.

The modest range of median diameter in the present experiment suggests that the atomization conditions produced similarly sized domains, whereas the broader span observed in EXP10 indicates greater heterogeneity and fusion. Circularity values of 0.556–0.599 show that the domains deviated from an ideal circle, while solidity values of 0.837–0.864 indicate that most domains remained comparatively compact despite folds and indentations. These metrics are highly useful for relative comparison among runs.

The high-magnification images provide an important process-quality contrast. EXP5, EXP13, EXP14, and EXP15 showed comparatively smoother surfaces and fewer pronounced defects, whereas EXP4, EXP7, EXP8, EXP10, and EXP12 showed more wrinkling, fissures, folds, and crater-like features. Deng et al. [21] similarly showed that wall material strongly changed aggregation, fractures, collapse, and surface morphology in spray-dried bitter-gourd powders, including formulations prepared with resistant starch. The present results extend that concept by showing that, even when the wall material is held constant, processing conditions can generate markedly different surface architectures.

EXP14 and EXP8 had a low water activity, and neither showed a resolvable low-temperature DSC endotherm, yet their surface characteristics differed: EXP14 had one of the highest circularity values and a comparatively smooth, low-defect morphology, whereas EXP8 showed a greater frequency of defects. Thus, strong drying in terms of water removal does not automatically yield the most favorable particle surface. This finding supports a multiresponse optimization strategy in which water activity, particle-size distribution, geometric compactness, and surface integrity are considered simultaneously rather than selecting a run on the basis of a single response.

## 5. Limitations

This study was conducted using mezcal vinasse obtained from a single distillery and agave source; therefore, the variability associated with different agave species, production practices, geographical origins, and seasonal conditions was not evaluated. Both the HMF-mitigation treatment and the spray-drying process were performed at laboratory scale, and the model-predicted optimal conditions require independent experimental validation before their reproducibility and scalability can be established. Phenolic preservation was evaluated as total phenolic retention, which does not identify compound-specific adsorption, oxidation, degradation, or transformation during treatment with activated carbon and laccase. FTIR, DSC, and SEM provided complementary information on matrix organization, water–matrix interactions, and particle morphology, but did not directly determine long-range crystallinity, glass-transition behavior, bioactive-compound localization, or storage stability. Finally, the present results do not include selective microbial fermentation or metabolite production.

## 6. Conclusions

The sequential treatment of the vinasse with lacasse and activated carbons at different pH and spray-drying approach demonstrated the feasibility of converting the mezcal by-product into a low-water-activity powder while reducing HMF and retaining part of its phenolic fraction. The treatment results highlight a central requirement for valorization, which is that HMF mitigation must be evaluated alongside phenolic preservation, because maximizing removal alone can substantially diminish the chemical fraction of potential interest. Within the experimental domain, treatment with 0.01 g activated carbon/100 mL and 873 µL laccase/100 mL at pH 4.5 provided the best observed compromise, achieving 76.69% HMF reduction and 72.30% phenolic retention. These conditions therefore constitute an experimentally supported starting point for further process development.

For spray drying with corn starch, an inlet temperature of 140 °C, an airflow setting of 50 V, and a feed rate of 10 mL/min provided the most favorable observed combination of water activity and particle morphology. The resulting powder had a water activity of 0.1306, comparatively smooth surfaces with few pronounced defects, and no resolvable low-temperature DSC endotherm. The complementary characterization showed that drying conditions influenced local matrix organization, water–matrix interactions, and particle architecture while preserving broadly similar FTIR functional-group profiles. Consequently, powder development should consider these properties together, since low water activity alone does not ensure favorable surface morphology.

Overall, this work establishes an experimental basis for the valorization of mezcal vinasse through coordinated HMF mitigation and powder formation. The selected experimental conditions provide practical candidates for subsequent validation, while storage stability, residual-compound safety, and biological functionality remain to be investigated.

## Figures and Tables

**Figure 1:**
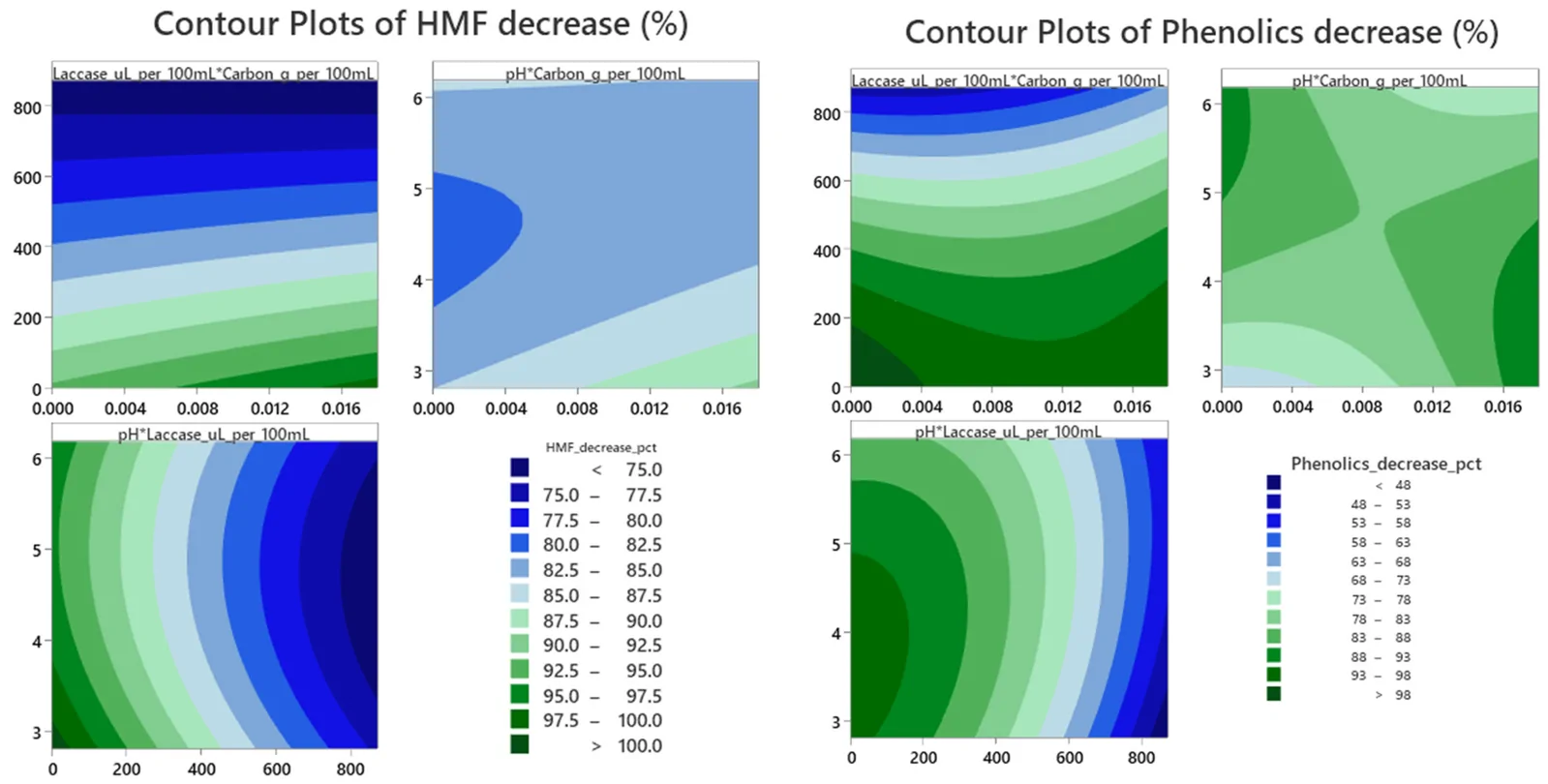
Contour plots of HMF decrease (%) and phenolics decrease (%).

**Figure 2:**
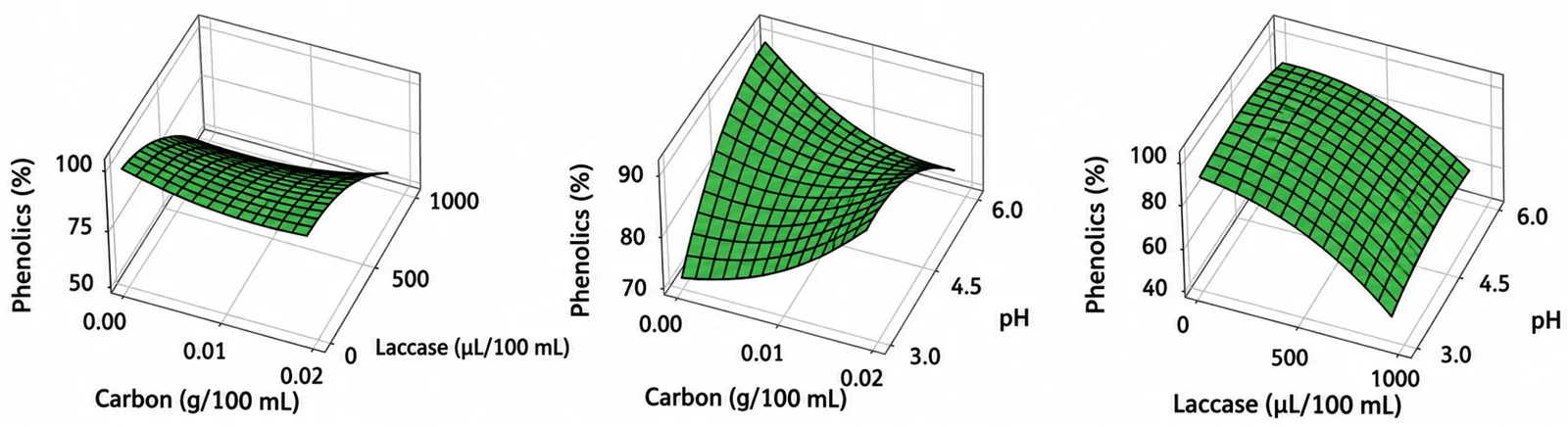
Surface plots for phenolic diminishment.

**Figure 3:**
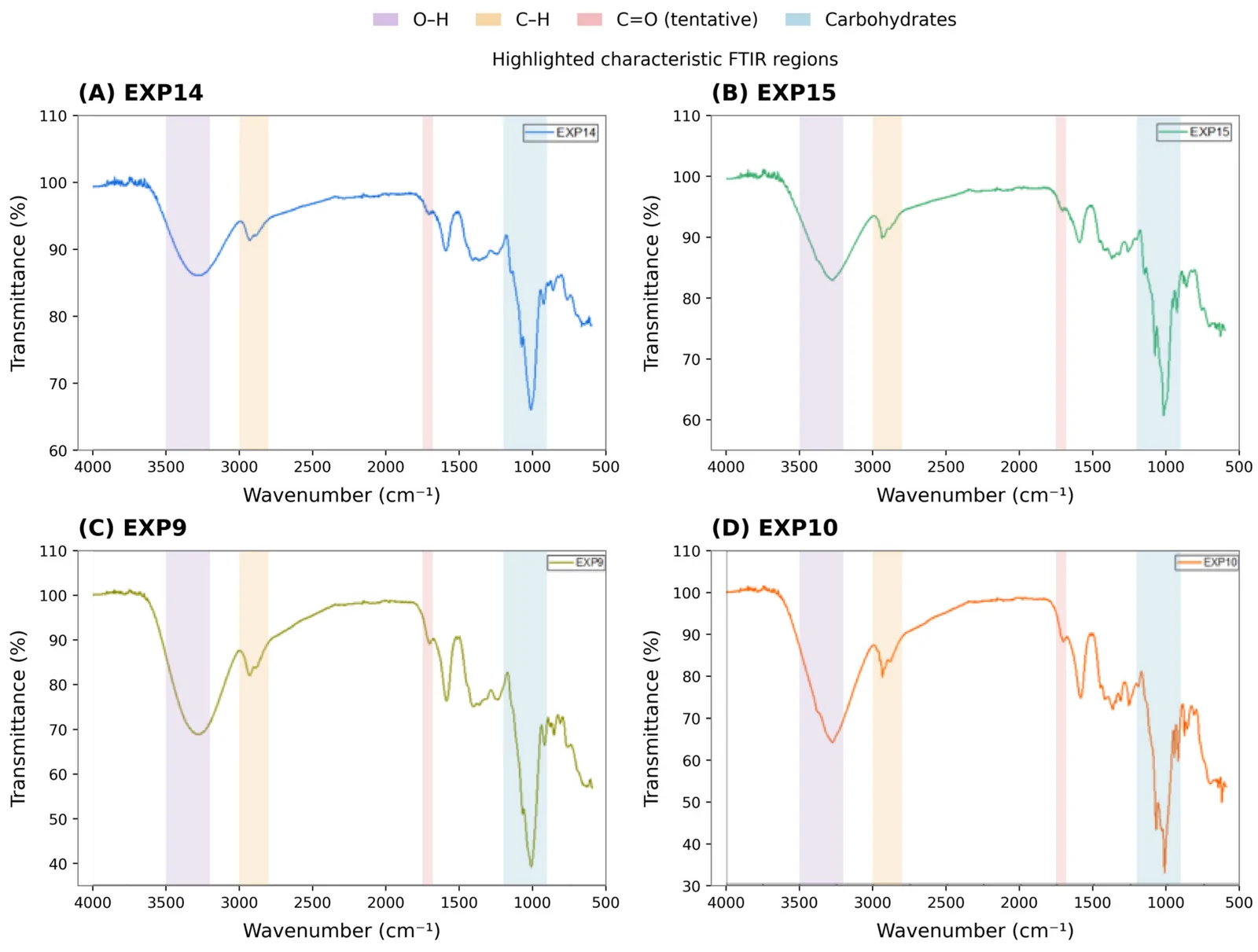
FTIR spectra of selected spray-dried starch–vinasse formulations: (**A**) EXP14, (**B**) EXP15, (**C**) EXP9, and (**D**) EXP10. Shaded regions highlight O–H stretching (3500–3200 cm^−1^), C–H stretching (3000–2800 cm^−1^), the region containing the tentatively assigned carbonyl-associated shoulder (1750–1680 cm^−1^), and carbohydrate-associated fingerprint absorptions (1200–900 cm^−1^).

**Figure 4:**
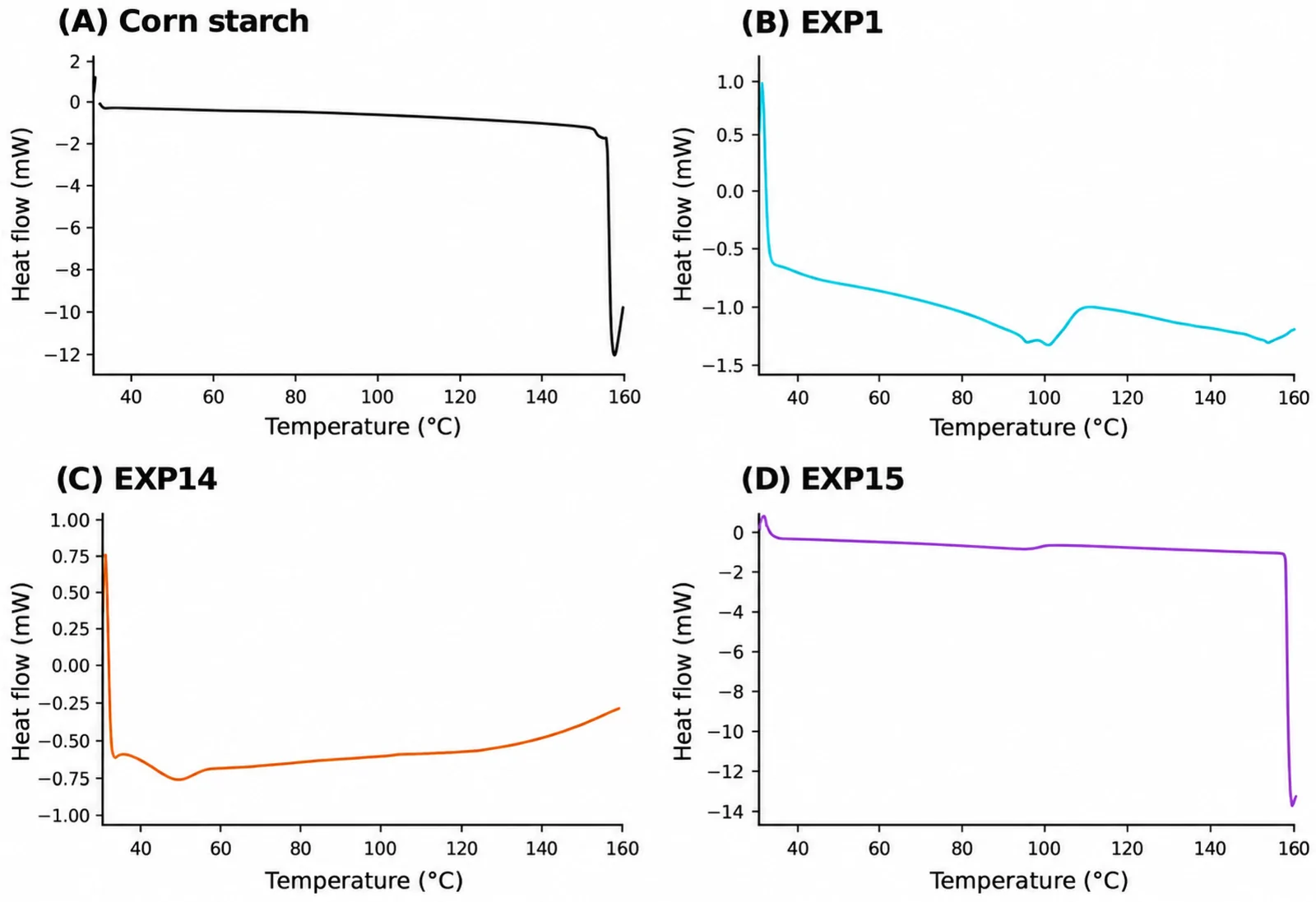
DSC thermograms of (**A**) corn starch, (**B**) EXP1, (**C**) EXP14, and (**D**) EXP15.

**Figure 5:**
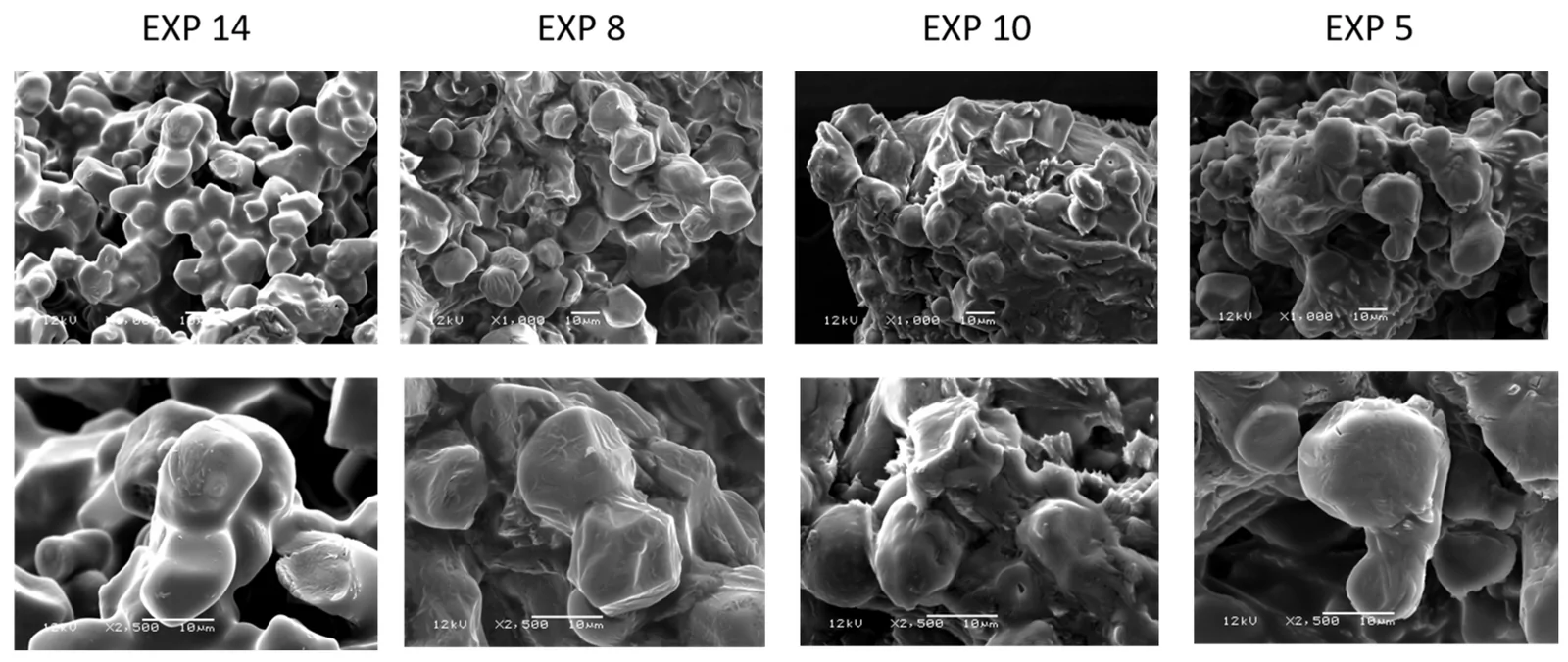
Representative SEM micrographs of powders produced under contrasting spray-drying conditions.

**Table 1:** Central Composite Design and Primary Responses.

Experiment	1	2	3	4	5	6	7	8	9	10	11	12	13	14	15	16	17	18	19	20
Activated carbon (g/100 mL)	0.01	0.01	0.02	0.01	0.01	0.01	0.01	0.02	0.01	0.01	0.01	0.01	0.01	0	0.02	0.02	0.01	0.02	0.01	0.01
Laccase (µL/100 mL)	175	698	698	175	436	436	698	436	873	436	436	436	436	436	175	175	436	698	0	436
pH	5.5	5.5	3.5	3.5	4.5	2.82	3.5	4.5	4.5	6.18	4.5	4.5	4.5	4.5	3.5	5.5	4.5	5.5	4.5	4.5
HMF reduction (%)	91.9	77.3	76.1	92.3	84.3	90	76.5	87.6	76.7	84.8	83.4	82.8	82.6	80.9	93.9	91.6	82.3	74.8	93.9	83
Phenolic retention (%)	5.46	14.1	13.5	11.2	20.7	24.9	34.9	28.8	72.3	42.6	27.4	18	11.4	18.8	4.46	3.35	9.61	13.3	2.92	9.76

**Table 2:** Box–Behnken design matrix.

Experiment	1	2	3	4	5	6	7	8	9	10	11	12	13	14	15
Inlet T (°C)	120	120	100	120	140	120	100	140	120	100	140	120	120	140	100
Air flow (V)	50	50	30	40	40	30	50	30	40	40	40	30	40	50	40
Solution flow (mL/min)	7	12	10	10	7	7	10	10	10	7	12	12	10	10	12

## Data Availability

The original contributions presented in this study are included in the article. Further inquiries can be directed to the corresponding author.

## Abbreviations

The following abbreviations are used in this manuscript:

ANOVA	Analysis of variance
BBD	Box–Behnken design
CCD	Central composite design
DSC	Differential scanning calorimetry
FTIR	Fourier-transform infrared spectroscopy
HMF	5-(hydroxymethyl)furfural
HPLC-DAD	High-performance liquid chromatography coupled to diode-array detection
PCA	Principal component analysis
RSM	Response surface methodology
SEM	Scanning electron microscopy
aw	Water activity
D10	10th percentile particle diameter
D50	Median particle diameter
D90	90th percentile particle diameter
